# Sp3 Transcription Factor Cooperates with the Kaposi’s Sarcoma-Associated Herpesvirus ORF50 Protein To Synergistically Activate Specific Viral and Cellular Gene Promoters

**DOI:** 10.1128/JVI.01143-20

**Published:** 2020-08-31

**Authors:** Li-Yu Chen, Lee-Wen Chen, Kuo-Ti Peng, Chien-Hui Hung, Pey-Jium Chang, Shie-Shan Wang

**Affiliations:** a Graduate Institute of Clinical Medical Sciences, College of Medicine, Chang-Gung University, Taoyuan, Taiwan; b Department of Respiratory Care, Chang-Gung University of Science and Technology, Chiayi, Taiwan; c Department of Pediatric Surgery, Chang-Gung Memorial Hospital, Chiayi, Taiwan; d Department of Orthopedic Surgery, Chang-Gung Memorial Hospital, Chiayi, Taiwan; e Department of Nephrology, Chang-Gung Memorial Hospital, Chiayi, Taiwan; f School of Medicine, Chang-Gung University, Taoyuan, Taiwan; Northwestern University

**Keywords:** IL-10, Kaposi’s sarcoma-associated herpesvirus, ORF21, ORF50, ORF56, ORF60, Sp3, synergistic activation

## Abstract

The Kaposi’s sarcoma-associated herpesvirus (KSHV)-encoded open reading frame 50 (ORF50) protein is the key transactivator responsible for the latent-to-lytic switch. Here, we investigated the transcriptional activation of the ORF56 gene (encoding a primase protein) by ORF50 and successfully identified an ORF50-responsive element located in the promoter region between positions −97 and −44 (designated 56p-RE). This 56p-RE element contains a noncanonical RBP-Jκ-binding sequence and a nonconsensus Sp1/Sp3-binding sequence. Electrophoretic mobility shift assays revealed that RBP-Jκ, Sp3, and ORF50 could form stable complexes on the 56p-RE element. Importantly, transient-reporter analysis showed that Sp3, but not RBP-Jκ or Sp1, acts in synergy with ORF50 to activate the 56p-RE-containing reporter construct, and the synergy mainly depends on the Sp1/Sp3-binding region of the 56p-RE element. Sequence similarity searches revealed that the promoters for ORF21 (thymidine kinase), ORF60 (ribonucleotide reductase, small subunit), and cellular interleukin-10 (IL-10) contain a sequence motif similar to the Sp1/Sp3-binding region of the 56p-RE element, and we found that these promoters could also be synergistically activated by ORF50 and Sp3 via the conserved motifs. Noteworthily, the conversion of the Sp1/Sp3-binding sequence of the 56p-RE element into a consensus high-affinity Sp-binding sequence completely lost the synergistic response to ORF50 and Sp3. Moreover, transcriptional synergy could not be detected through other ORF50-responsive elements from the viral PAN, K12, ORF57, and K6 promoters. Collectively, the results of our study demonstrate that ORF50 and Sp3 can act in synergy on the transcription of specific gene promoters, and we find a novel conserved *cis*-acting motif in these promoters essential for transcriptional synergy.

**IMPORTANCE** Despite the critical role of ORF50 in the KSHV latent-to-lytic switch, the molecular mechanism by which ORF50 activates its downstream target genes, especially those that encode the viral DNA replication enzymes, is not yet fully understood. Here, we find that ORF50 can cooperate with Sp3 to synergistically activate promoters of the viral ORF56 (primase), ORF21 (thymidine kinase), and ORF60 (ribonucleotide reductase) genes via similar Sp1/Sp3-binding motifs. Additionally, the same synergistic effect can be seen on the promoter of the cellular IL-10 gene. Overall, our data reveal an important role for Sp3 in ORF50-mediated transactivation, and we propose a new subclass of ORF50-responsive elements in viral and cellular promoters.

## Abstract (precis)

Despite the critical role of ORF50 in the KSHV latent-to-lytic switch, the molecular mechanism by which ORF50 activates its downstream target genes, especially those that encode the viral DNA replication enzymes, is not yet fully understood. Here, we find that ORF50 can cooperate with Sp3 to synergistically activate promoters of the viral ORF56 (primase), ORF21 (thymidine kinase), and ORF60 (ribonucleotide reductase) genes via similar Sp1/Sp3-binding motifs. Additionally, the same synergistic effect can be seen on the promoter of the cellular IL-10 gene. Overall, our data reveal an important role for Sp3 in ORF50-mediated transactivation, and we propose a new subclass of ORF50-responsive elements in viral and cellular promoters.

## INTRODUCTION

Kaposi’s sarcoma-associated herpesvirus (KSHV), also referred to as human herpesvirus 8 (HHV-8), is etiologically associated with at least three malignancies, including Kaposi’s sarcoma (KS), primary effusion lymphoma (PEL), and multicentric Castleman’s disease (MCD) (1–3). Similar to other herpesviruses, KSHV displays two distinctive life cycles, latency and the lytic cycle (4, 5). In addition to maintaining the long-term persistence of the virus, both the latent and lytic cycles are also considered critical for the pathogenesis of KSHV-associated diseases (6–9). Although the authentic physiological stimuli that determine the switch between latency and the lytic cycle are not yet fully understood, different chemical agents or biological conditions have been reported to induce KSHV reactivation in cultured PEL cell lines, such as sodium butyrate (SB), 12-*O*-tetradecanoylphorbol-13-acetate (TPA), specific proinflammatory cytokines, hypoxia, endoplasmic reticulum stress, reactive oxygen species, and high glucose levels (4, 10–16).

Upon KSHV reactivation from latency, viral lytic genes are expressed in a cascade fashion, i.e., immediate early (IE), early, and late gene expression, which ultimately leads to the production of mature and infectious virions (17, 18). Ample evidence has shown that the IE gene product encoded by open reading frame 50 (ORF50) of the viral genome is the key regulator for the switch from latency to the lytic cycle (18–20). The ectopic expression of the ORF50 protein, also known as RTA (replication and transcription activator), in latently KSHV-infected cells is sufficient to trigger the lytic cascade to completion. The ORF50 protein is a 691-amino-acid (aa) transcriptional activator that contains a basic DNA-binding domain (aa 1 to 390) and an acidic activation domain (aa 486 to 691) (21–23). Transient-transfection experiments demonstrated that the ORF50 protein is capable of promoting the transcriptional activation of a group of viral lytic gene promoters. These include promoters for ORF50 itself, polyadenylated nuclear (PAN) RNA, K12 (kaposin), oriLyt-associated transcript (oriLyt-T), ORF57, K6 (viral macrophage inflammatory protein 1 [vMIP-1]), K8 (KSHV-encoded basic region-leucine zipper protein [K-bZIP]), K9 (viral interferon regulatory factor 1 [vIRF1]), ORF21 (thymidine kinase), K2 (viral interleukin-6 [vIL6]), K3 (modulator of immune recognition 1 [MIR1]), K5 (MIR2), ORF6 (single-stranded DNA-binding protein), ORF59 (polymerase processivity factor), K14 (viral orexin-2 [vOX-2]), ORF74 (viral G protein-coupled receptor [vGPCR]), ORF8 (glycoprotein B), ORF60 (ribonucleotide reductase, small subunit), ORF61 (ribonucleotide reductase, large subunit), ORF70 (thymidylate synthase), ORF19 (a tegument protein), ORF47 (glycoprotein L), ORF46 (uracil DNA glycosylase), and ORF45 (a tegument protein) (10, 24–43).

Currently, there are two well-characterized molecular mechanisms by which ORF50 activates its downstream target promoters during the lytic cycle. One mechanism is mediated by the direct binding of ORF50 to target DNA, and the other is operated by the indirect access of ORF50 to DNA via the interaction with cellular transcription factors already bound to DNA (20). The PAN, K12, and oriLyt-T gene promoters are the best-known direct targets of ORF50 (24–26). The ORF50-responsive elements (ORF50 REs) in these promoters share highly conserved nucleotides, AAATGGGTGGCTAACCCCTACATAA (24–26), which are essential for the direct binding of ORF50. In contrast, most of the identified ORF50-responsive genes actually lack the direct ORF50-binding motif in their promoters. The activation of each of these promoters by ORF50 is mainly dependent on the interaction of ORF50 with other DNA-bound transcription factors such as RBP-Jκ (also known CSL or CBF-1), C/EBPα, Oct-1, or Stat3 (35, 40, 42, 44, 45). Among them, the DNA-binding protein RBP-Jκ, a key effector of the Notch signaling pathway (46), is the most well-characterized coregulator critically involved in the ORF50-mediated transactivation of viral lytic genes. Typically, RBP-Jκ functions as a transcriptional repressor on target gene promoters under normal conditions; however, the interaction of ORF50 with RBP-Jκ could cause the conversion of the RBP-Jκ complex from a repressor to an activator of target gene promoters. We and others have demonstrated that RBP-Jκ plays an essential role in the ORF50-mediated transactivation of a number of viral lytic gene promoters such as the ORF57, K6, ORF61, ORF47, ORF46, and ORF45 promoters (26–30, 40, 43). Despite extensive studies, it remains possible that ORF50 may use different mechanisms to transcriptionally regulate the other target gene promoters.

The transcription factors Sp1 and Sp3 are members of the specificity protein/Kruppel-like factor (Sp/KLF) family, which are ubiquitously expressed in all mammalian cells and are involved in a variety of cellular processes (47–49). Both Sp1 and Sp3 share more than 90% sequence homology in their C-terminal DNA-binding domains (with three Cys_2_-His_2_ zinc fingers) and bind to the same cognate DNA elements containing the GC-rich core motif. Although Sp1 and Sp3 may recognize the same DNA element with similar binding affinities, the transcriptional functions of these two proteins are often not equivalent (47, 48). Despite evidence that Sp3 can act as a transcriptional activator, most studies have revealed that Sp3 usually functions as a transcriptional repressor in cells. Increasing evidence has also shown that Sp1 and Sp3 are expressed at higher levels in a number of cancer cells than in normal cells, and knockdown of Sp1 or Sp3 in cancer cells dramatically reduced tumorigenic and metastatic phenotypes (50). Thus, dysregulation of Sp1 and Sp3 may cause abnormal cell proliferation, differentiation, survival, migration, or invasion. Due to the fact that putative Sp1/Sp3-binding sites are widely distributed within the KSHV genome, it is possible that Sp1 or Sp3 may play critical roles in the regulation of viral gene expression.

In this study, we aimed to characterize the transcriptional regulation of the ORF56 gene that encodes a viral primase. Our results showed that the ORF56 promoter (ORF56p) could be activated by ORF50 independently of other viral factors, and an ORF50 RE in the promoter was mapped to the region from positions −97 to −44. This 56p-RE motif possesses a noncanonical RBP-Jκ-binding site and a nonconsensus Sp1/Sp3-binding site. Particularly, we found that Sp3, but not RBP-Jκ or Sp1, cooperated with ORF50 to synergistically activate the 56p-RE-containing reporter construct. Besides the ORF56 promoter, the promoters of the viral ORF21 and ORF60 genes as well as the cellular interleukin-10 (IL-10) gene, which contain sequence motifs similar to the 56p-RE element, could also be synergistically activated by ORF50 and Sp3. Based on our findings, here, we propose a molecular model for the transcriptional synergy operated by ORF50 and Sp3 at these promoters.

## RESULTS

### Transcriptional mapping and deletion analysis of the ORF56 promoter.

**FIG 1:**
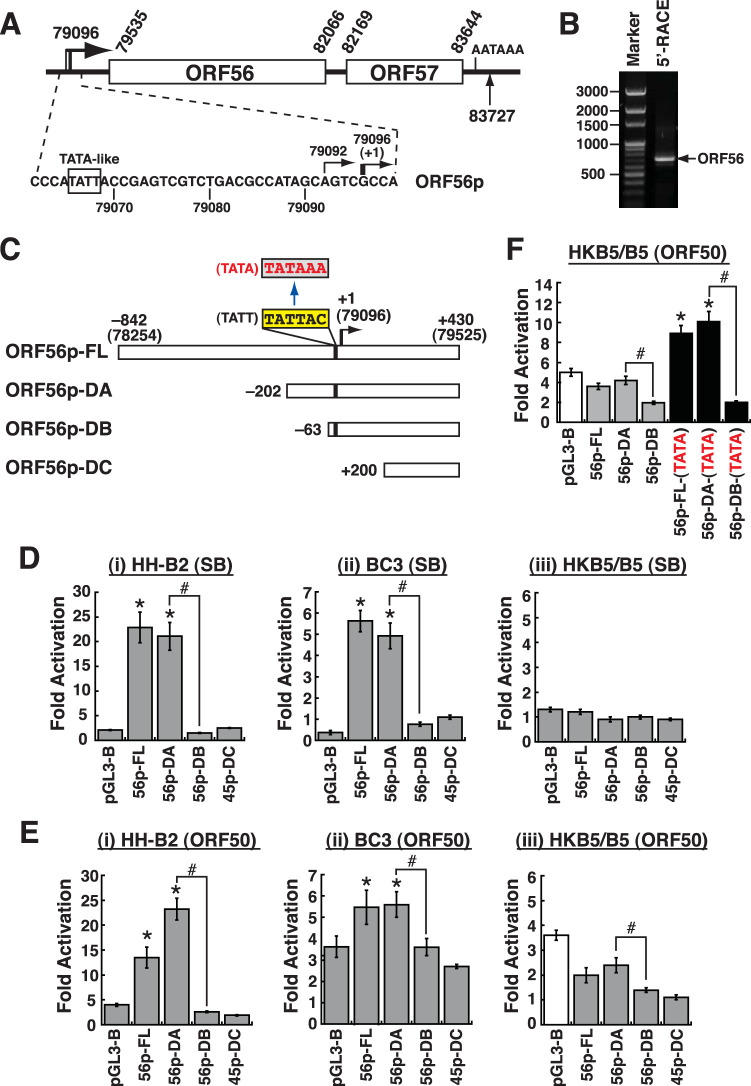
Mapping the transcriptional start sites of the ORF56 transcripts and defining the regulatory elements in the ORF56 promoter. (A) Diagram of the ORF56/ORF57 gene structure and the ORF56 core promoter region. The numbers in the diagram are the nucleotide positions of the KSHV genome (GenBank accession no. NC_009333). A noncanonical TATA box motif, TATT, is located upstream of the transcriptional start sites of the ORF56 gene. (B) Representative 5′-RACE analysis of ORF56 transcripts expressed in sodium butyrate-induced HH-B2 cells. (C) Diagram of reporter constructs containing a full-length ORF56 promoter (ORF56p-FL) and its 5′ deletions. Point mutations at the TATT box motif of the ORF56 promoter, from 5′-TATTAC to 5′-TATAAA (consensus TATA box), are shown in the diagram. (D) Responsiveness of the ORF56p reporter constructs to sodium butyrate (SB) in HH-B2, BC3, and HKB5/B5 cells. Forty-eight hours after transfection and treatment, cells were harvested, and luciferase assays were performed. Data are indicated as fold activation of the luciferase activity of the reporter construct in the presence of SB treatment over the activity in the absence of SB treatment. (E) Responsiveness of the ORF56p reporter constructs to ORF50 overexpression in HH-B2, BC3, and HKB5/B5 cells. Forty-eight hours after transfection, cells were harvested, and luciferase assays were performed. Data are presented as fold activation of the luciferase activity of the reporter construct in the presence of the ORF50 expression vector over the activity in the presence of the empty vector control. For HKB5/B5 cells, it should be noted that all ORF56p-driven reporter constructs did not show higher ORF50 responsiveness than the control reporter pGL3-B (white bar). (F) Transcriptional activation of the indicated ORF56p reporter constructs by ORF50 in HKB5/B5 cells. The reporter constructs, including ORF56p-FL-(TATA), ORF56p-DA-(TATA), and ORF56p-DB-(TATA), contain a consensus TATA box motif (5′-TATAAA). *, *P < *0.05, for results compared to those with pGL3-B; #, *P < *0.05, for results compared to those with the indicated reporter constructs.

The KSHV ORF56 gene encodes a primase protein that is essential for viral DNA replication. To study the transcriptional regulation of ORF56p, we first determined its transcriptional start site by 5′ RACE (rapid amplification of cDNA ends). In this experiment, total RNAs that were extracted from SB-treated HH-B2 cells, a KSHV-positive PEL cell line, were used as the starting material for RACE analysis. Results from RACE analysis revealed that two closely clustered transcriptional start sites, at nucleotide (nt) 79096 (major) and nt 79092 (minor), were mapped to the ORF56 transcripts (Fig. 1A and B). Upstream of the identified transcriptional start sites, a noncanonical TATA box, TATT, was found in the ORF56p region (Fig. 1A). To further determine the critical elements responsible for ORF56p activation during viral lytic replication, the promoter region from positions −842 to +430 was cloned into pGL3-Basic, a luciferase reporter vector (Fig. 1C). In the transactivation experiments, the resultant full-length ORF56p (ORF56p-FL) reporter construct was transfected into KSHV-positive cell lines, including HH-B2 and BC3, and the transfected cells were then untreated or treated with SB. Forty-eight hours after transfection and treatment, we found that the luciferase activity of the ORF56p-FL reporter construct was markedly activated by SB compared to that of pGL3-Basic (Fig. 1Di and ii). However, the ORF56p-FL reporter construct could not be activated by SB in a KSHV-negative cell line, HKB5/B5 (Fig. 1Diii). These results suggested that specific viral factors in HH-B2 and BC3 cells could play essential roles in the SB-mediated activation of the ORF56p-FL reporter construct. Moreover, deletion analysis of the promoter revealed that a 5′ deletion to position −202, deletion A (ORF56p-DA), still retained the full response to SB in HH-B2 or BC3 cells (Fig. 1Di and ii). In contrast, a further 5′ deletion to position −63 (ORF56p-DB) or to position +200 (ORF56p-DC) in the promoter dramatically abolished the response to SB in HH-B2 or BC3 cells (Fig. 1Di and ii). These results indicated that the promoter region from positions −202 to −63 was necessary for SB-mediated transcriptional activation (Fig. 1D).

Since ORF50 is a key regulator of KSHV reactivation, the effects of ORF50 on the ORF56p activity in HH-B2, BC3, or HKB5/B5 cells were also determined (Fig. 1E). Similar to SB treatment, cotransfection of the ORF50 expression plasmid in HH-B2 or BC3 cells for 48 h significantly activated the luciferase activity of the ORF56p-FL reporter construct compared to pGL3-Basic. Furthermore, deletion analysis showed that the ORF56p region from positions −202 to −63 was required for ORF50 responsiveness in HH-B2 and BC3 cells (Fig. 1Ei and ii). In HKB5/B5 cells, although the ORF56p-FL reporter construct did not show higher ORF50 responsiveness than the control reporter pGL3-Basic, we noticed that both the ORF56p-FL and ORF56p-DA reporter constructs substantially produced higher ORF50 responsiveness than the ORF56p-DB and ORF56p-DC reporter constructs (Fig. 1Eiii). Due to the lack of a canonical TATA box in the ORF56 promoter, we suspected that the unusual TATT motif in the core promoter might affect the overall promoter activity and weaken ORF50 responsiveness in KSHV-negative cells. To test this possibility, we converted the TATT motif (5′-TATTAC) into a consensus TATA box motif (5′-TATAAA) in the full-length ORF56 promoter and its deletion mutants, leading to the generation of the ORF56p-FL-(TATA), ORF56p-DA-(TATA), and ORF56p-DB-(TATA) reporter constructs (Fig. 1C and F). We found that the ORF56p-FL-(TATA) and ORF56p-DA-(TATA) reporter constructs consistently displayed higher ORF50 responsiveness than pGL3-Basic in HKB5/B5 cells (Fig. 1F). In contrast, the ORF56p-DB-(TATA) reporter construct that lacks the ORF50-responsive region was not responsive to ORF50 in HKB5/B5 cells (Fig. 1F). These results demonstrated that the ORF56 promoter is a downstream target of ORF50, and an ORF50-responsive region could be located between positions −202 and −63 in the promoter.

### Fine mapping of the SB- or ORF50-responsive element in the ORF56 promoter.

**FIG 2:**
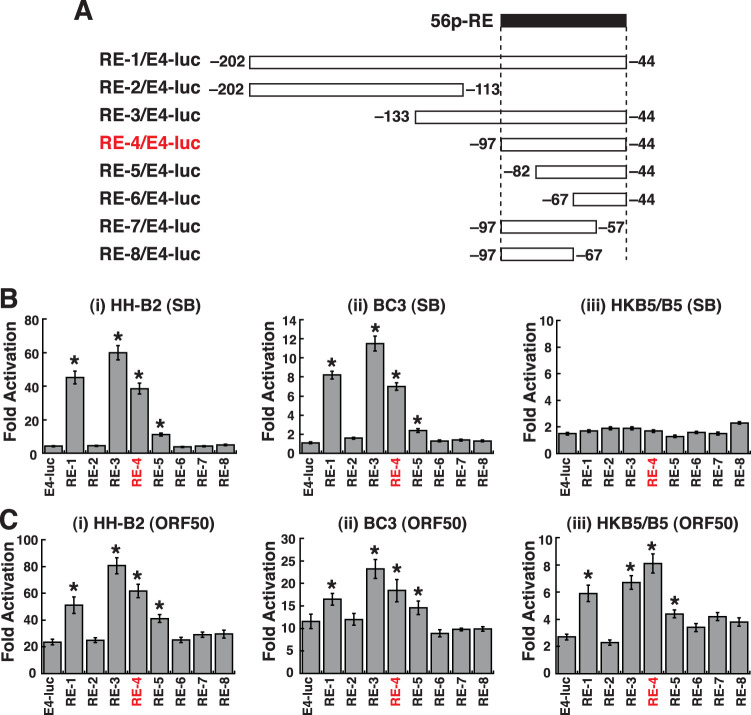
Mapping the minimal ORF50-responsive element in the ORF56 promoter. (A) Schematic diagram of a series of ORF56p regions in the reporter plasmid pE4-luc. An identified ORF50-responsive element located from positions −97 to −44, named 56p-RE, is shown at the top of the diagram. (B) Transcriptional activation of the indicated reporter constructs by SB in HH-B2, BC3, and HKB5/B5 cells. Data are indicated as fold activation of the luciferase activity of the reporter construct in the presence of SB treatment over the activity in the absence of SB treatment. (C) Transcriptional activation of the indicated reporter constructs by ORF50 in HH-B2, BC3, and HKB5/B5 cells. Data are presented as fold activation of the luciferase activity of the reporter construct in the presence of the ORF50 expression vector over the activity in the presence of the control vector. *, *P < *0.05, for results compared to those with pE4-luc.

To further map a minimal SB-responsive element or ORF50 RE in the ORF56 promoter, the promoter region from positions −202 to −44, which probably covered all necessary *cis*-regulatory elements, was transferred to pE4-luc, a luciferase reporter plasmid carrying a minimal adenovirus E4 promoter (Fig. 2A). Compared to pE4-luc, the RE-1/E4-luc construct with the ORF56p(−202/−44) region produced a high-level response to SB in HH-B2 or BC3 cells but not in HKB5/B5 cells (Fig. 2B). These results indicated that the ORF56p(−202/−44) region sufficiently conferred a response to SB in KSHV-positive cell lines. A series of 5′ or 3′ deletions of the ORF56p(−202/−44) region were subsequently cloned into pE4-luc to map the minimal responsive element to SB. After the transfection of these reporter constructs into HH-B2 or BC3 cells, we found that an important SB-responsive element was located at the ORF56p region from positions −97 to −44 (Fig. 2A and B, RE-4). For identifying the ORF50 RE in the ORF56 promoter, the same reporter constructs as the ones described above were individually cotransfected with the ORF50 expression plasmid in HH-B2, BC3, or HKB5/B5 cells. As expected, the RE-1/E4-luc reporter construct demonstrated a significant response to ORF50 in HH-B2, BC3, and HKB5/B5 cells (Fig. 2C). Importantly, the patterns of activation of the deleted ORF56p reporter constructs by ORF50 in all tested cell lines were similar (Fig. 2C), and a functional ORF50 RE was mapped to the promoter region from positions −97 to −44 (Fig. 2C, RE-4). The ORF56p(−97/−44) element was selected for further characterization and was designated “56p-RE” in this study.

### Potential cellular factors involved in the activation of the ORF56 promoter by ORF50.

**FIG 3:**
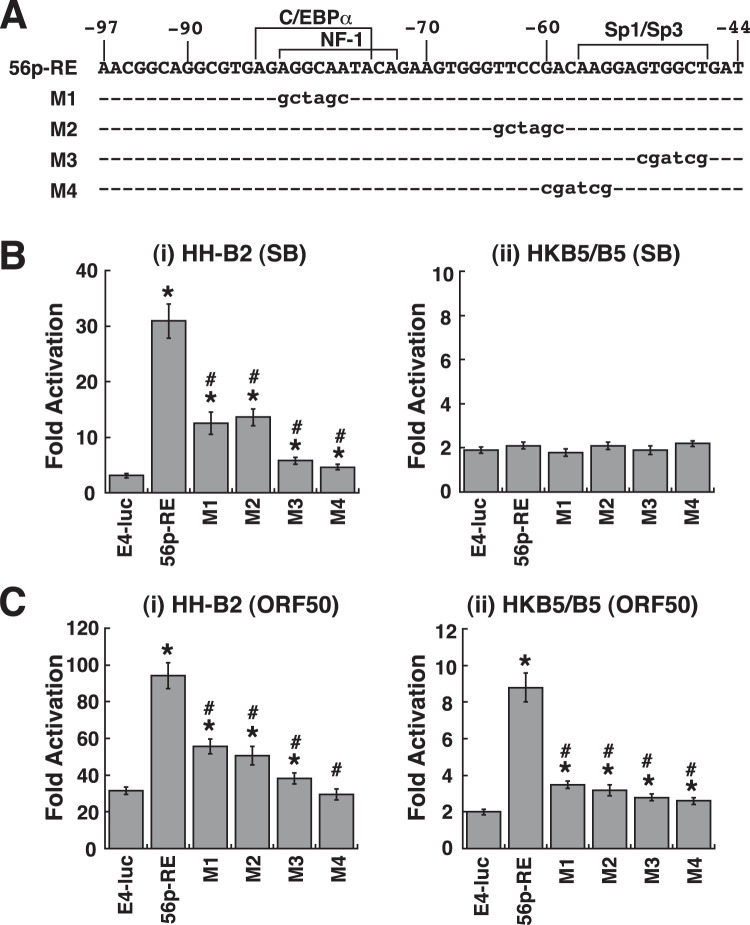
Point mutational analysis of the 56p-RE element. (A) DNA sequences of the wild-type and mutant 56p-RE elements in pE4-luc. The 56p-RE element was predicted to have binding sites for transcription factors, including C/EBPα, NF1, and Sp1/Sp3. (B) Transcriptional activation of the reporter constructs containing the wild-type or mutant 56p-RE elements (M1 to M4) by SB in HH-B2 and HKB5/B5 cells. (C) Transcriptional activation of the indicated reporter constructs by ORF50 in HH-B2 and HKB5/B5 cells. *, *P < *0.05, for results compared to those with pE4-luc; #, *P < *0.05, for results compared to those with the wild-type 56p-RE reporter construct.

Sequence analysis of the 56p-RE element failed to find a direct ORF50-binding motif; however, at least three binding sites for transcription factors, including C/EBPα, NF1, and Sp1/Sp3, were predicted (Fig. 3A). To analyze the potential role of these binding sites in the response to SB or ORF50, point mutations were introduced into the 56p-RE element (M1 to M4) (Fig. 3A). Transient transfection of these constructed reporter plasmids into HH-B2 cells revealed that mutations at these predicted binding sites or outside the binding sites in the 56p-RE element resulted in reduced responses to SB treatment or to ORF50 overexpression in HH-B2 cells (Fig. 3B and Ci). In HKB5/B5 cells, all mutated reporter constructs (M1 to M4) also showed reduced ORF50 responsiveness (Fig. 3Cii). These results suggested that the entire 56p-RE element was critical for supporting full ORF50 responsiveness.

**FIG 4:**
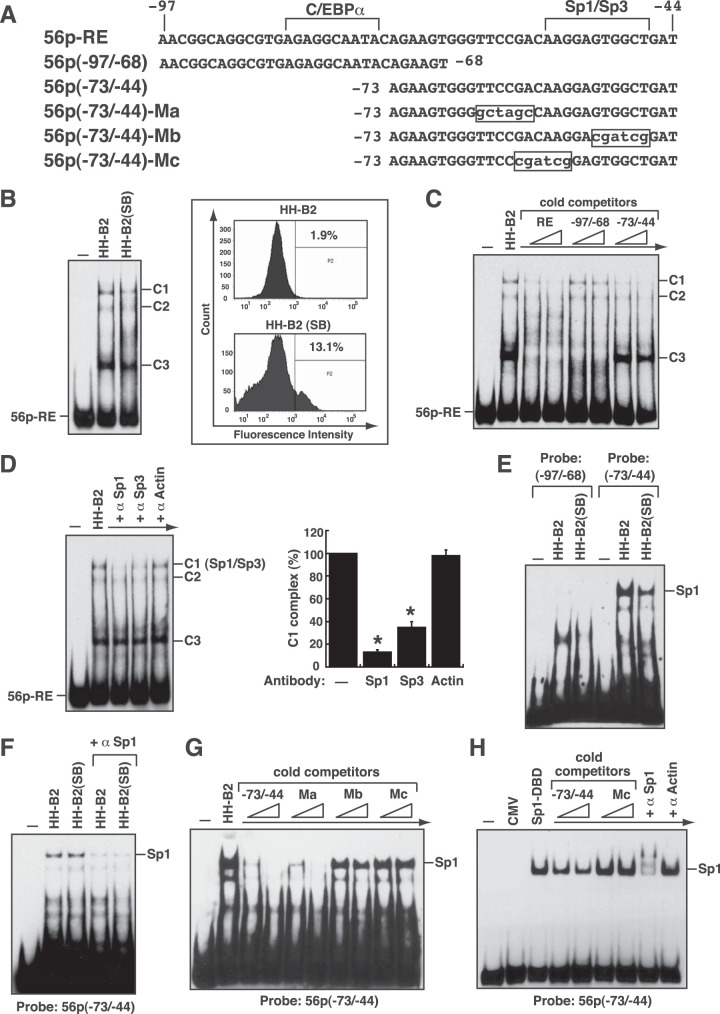
Sp1 specifically binds to the 3′ region of the 56p-RE motif. (A) DNA sequences of the 56p-RE element and its mutants. A predicted Sp1/Sp3-binding site is located in the 3′ region of the 56p-RE element. (B) EMSAs using untreated or SB-treated HH-B2 cell lysates. The 56p-RE element labeled with biotin was used as the probe in EMSAs. Three major protein/DNA complexes (C1, C2, and C3) were detected in EMSAs using the total cell lysate from untreated or SB-treated HH-B2 cells. For analyzing lytic induction in untreated or SB-treated HH-B2 cells, the percentage of ORF50-positive cells was determined by flow cytometry analysis using anti-ORF50 antibody (right). (C) Binding competition analysis in EMSAs. EMSA was performed using the total HH-B2 cell lysate, and different cold competitors, including the 56p-RE, 56p(−97/−68) and 56p(−73/−44) elements, were added at a concentration of a 30- or 50-fold molar excess relative to the labeled probe. (D) Antibody supershift or blockade assay by an EMSA. For the supershift/blocking assay, specific antibodies against Sp1, Sp3, or actin were added to EMSA reaction mixtures, and the intensities of the C1 complex remaining in the gel were quantified by densitometry (right). *, *P < *0.05, for results compared to those in the absence of antibody (*n *= 3). (E) EMSA using the 56p(−97/−68) or 56p(−73/−44) element as a probe. A putative Sp1/DNA complex was indicated by an EMSA using the 56p(−73/−44) probe. (F) Antibody supershift or blockade assay by an EMSA using the 56p(−73/−44) probe. The anti-Sp1 antibody was used in the supershift/blocking test. (G) Binding competition analysis by an EMSA with the 56p(−73/−44) probe. Cold competitors, including the 56p(−73/−44) element and its mutants Ma, Mb, and Mc shown in panel A, were added at a concentration of a 30- or 50-fold molar excess relative to the labeled probe. (H) Specific binding of the Sp1 DNA-binding domain (Sp1-DBD) to the 56p(−73/−44) element. Total protein lysates of 293T cells that were transfected with the plasmid expressing the Sp1-DBD (aa 300 to 785) were used in an EMSA. Both the binding competition test and the antibody supershift/blocking test were included in the EMSA. CMV, cytomegalovirus.

**FIG 5:**
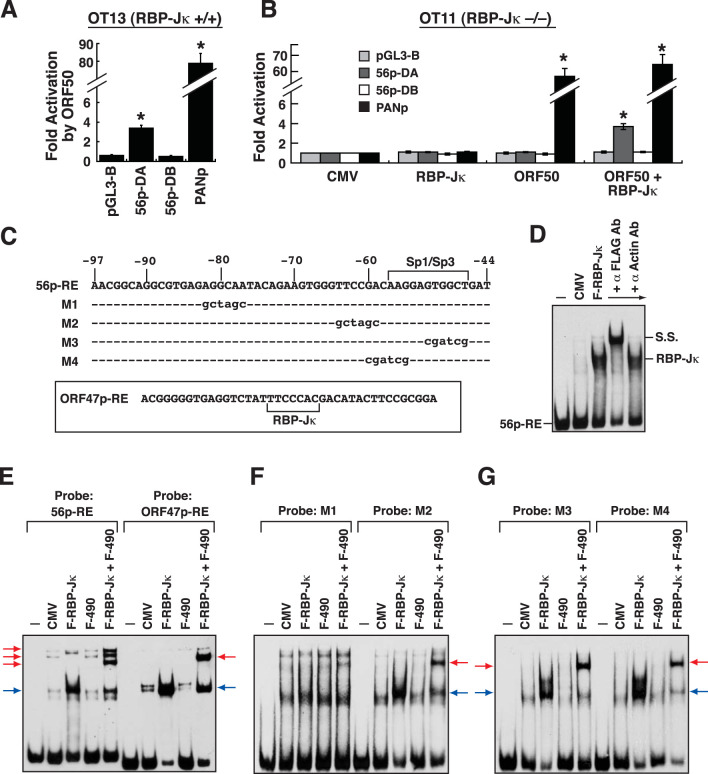
RBP-Jκ is involved in ORF50-mediated ORF56p transactivation. (A) Activation of the ORF56p reporter constructs by ORF50 in OT13 cells. The ORF56p-DA and ORF56p-DB reporter constructs were included in the experiment, and the PANp(−1875/+34) reporter construct served as a positive control. (B) Effects of RBP-Jκ, ORF50, or both RBP-Jκ and ORF50 on the activation of the ORF56p reporter constructs in RBP-Jκ-deficient OT11 cells. (C) DNA sequences of the wild-type and mutant 56p-RE elements as well as the ORF50 RE from the ORF47 promoter (designated ORF47p-RE). The ORF47p-RE element contains a consensus RBP-Jκ-binding site. (D) EMSA showing the binding of RBP-Jκ to the 56p-RE probe. Total protein lysates of 293T cells that were transfected with the plasmid expressing FLAG-tagged RBP-Jκ (F-RBP-Jκ) were used in the EMSA. For the supershift assay, anti-FLAG antibody (Ab) was added to EMSA reaction mixtures. S.S., supershifted complex. (E) EMSAs using the 56p-RE or ORF47p-RE element as a probe. Protein extracts of 293T cells that were transfected with the F-RBP-Jκ expression plasmid, the F-ORF50(1–490) expression plasmid, or both expression plasmids were used in EMSAs. The F-ORF50(1–490) expression plasmid encodes the FLAG-tagged ORF50 DNA-binding domain (aa 1 to 490) (F-490). Notably, several protein/DNA complexes corresponding to the F-RBP-Jκ/DNA complex (blue arrows) or F-RBP-Jκ/F-ORF50(1–490)/DNA complexes (red arrows) were observed in EMSAs. (F and G) EMSAs using mutant 56p-RE motifs (M1, M2, M3, and M4) as probes. 293T cell lysates containing F-RBP-Jκ, F-ORF50(1–490), or both F-RBP-Jκ and F-ORF50(1–490) were used in EMSAs. Blue arrows represent the location of the F-RBP-Jκ/DNA complex, whereas red arrows indicate the location of the F-RBP-Jκ/F-ORF50(1–490)/DNA complexes.

To investigate whether viral or cellular proteins specifically bound to the 56p-RE element, an electrophoretic mobility shift assay (EMSA) was performed using the protein lysates of HH-B2 cells that were untreated or treated with SB for 24 h. Compared to the use of the untreated HH-B2 cell lysate, the SB-treated cell lysate used in EMSAs did not show extra protein complexes on the 56p-RE probe (Fig. 4A and B). The failure to find additional protein/DNA complexes with the use of the SB-treated cell lysate in EMSAs could be due to a low proportion of cells undergoing lytic induction in HH-B2 cells after SB treatment, as shown by flow cytometry analysis using anti-ORF50 antibody (13.1% versus 1.9%) (Fig. 4B, right), or due to weak interactions between the probe DNA and the induced proteins under our EMSA conditions. There were three major protein/DNA complexes (C1, C2, and C3) detected in EMSAs using either the untreated or SB-treated HH-B2 cell lysate (Fig. 4B). To determine the binding specificity of these protein complexes, a binding competition assay was performed using the 56p-RE, 56p(−97/−68), and 56p(−73/−44) elements as cold competitors in EMSAs (Fig. 4A and C). The results showed that the C1 and C2 complexes seemed to be formed on the 3′ portion of the 56p-RE probe, whereas the C3 complex was formed on the 5′ portion of the 56p-RE probe. Since an Sp1/Sp3-binding site was predicted near the 3′ end of the 56p-RE element, antibodies specific to Sp1 or Sp3 were used in EMSAs to supershift or remove the potential protein/DNA complex. The addition of anti-Sp1 or anti-Sp3 antibody to EMSA reaction mixtures substantially removed the C1 complex, indicating that the C1 complex was an Sp1/DNA or Sp3/DNA complex (Fig. 4D). Since the Sp1/DNA complex was preferentially detected in the EMSA, we examined whether Sp1 indeed bound to the 3′ region of the 56p-RE element. Two partially overlapping DNA probe elements, 56p(−97/−68) and 56p(−73/−44), were then used in EMSAs (Fig. 4A and E). In combination with the antibody supershift/blocking assay, we showed that Sp1 specifically bound to the 56p(−73/−44) element in EMSAs (Fig. 5F). By using the wild-type 56p(−73/−44) element or its mutants (Ma, Mb, and Mc) as cold competitors, we further confirmed that Sp1 bound to the predicted Sp1/Sp3-binding site within the 56p(−73/−44) element (Fig. 4A and G, Mb and Mc). Finally, an EMSA was performed using the protein extract of 293T cells that overexpressed the Sp1 DNA-binding domain (Sp1-DBD) (aa 300 to 785). As shown in Fig. 4H, specific binding of the Sp1-DBD to the 56p(−73/−44) element was demonstrated by both the binding competition test and the supershift test in EMSAs.

### Role of RBP-Jκ in the ORF50-mediated activation of the ORF56 promoter.

Although there was no consensus binding site for RBP-Jκ in the ORF56 promoter, we sought to investigate whether the activation of the ORF56 promoter by ORF50 required RBP-Jκ. The ORF56p-DA and ORF56p-DB reporter constructs shown in Fig. 1C were individually cotransfected with the ORF50 expression plasmid into a mouse embryonic fibroblast cell line, OT13, or an RBP-Jκ knockout cell line, OT11 (Fig. 5A and B). In these experiments, the reporter construct containing the PAN promoter served as a positive control (Fig. 5A and B). Notably, the ORF56p-DA reporter construct that contains a functional ORF50 RE could be activated by ORF50 in OT13 cells (Fig. 5A) but not in OT11 cells (Fig. 5B). The ORF56p-DB reporter construct that lacks the ORF50 RE failed to be activated by ORF50 in either OT13 or OT11 cells (Fig. 5A and B). Importantly, we found that the cotransfection of the RBP-Jκ expression plasmid substantially restored the ORF50-mediated activation of the ORF56p-DA reporter construct in OT11 cells (Fig. 5B). These results suggested that RBP-Jκ is involved in the activation of the ORF56 promoter by ORF50.

To investigate whether RBP-Jκ directly bound to the 56p-RE element, the protein extract of 293T cells transfected with a plasmid expressing FLAG-tagged RBP-Jκ (F-RBP-Jκ) was used in EMSAs. Our data revealed that F-RBP-Jκ specifically bound to the 56p-RE element, and the protein/DNA complex formed in EMSAs could be supershifted by anti-FLAG antibody (Fig. 5C and D). However, when the protein lysate of 293T cells expressing the FLAG-tagged ORF50 DNA-binding domain (aa 1 to 490) [F-ORF50(1–490)] was used in EMSAs, we could not detect the binding of F-ORF50(1–490) to the 56p-RE element (Fig. 5E). Interestingly, compared to the use of the protein lysate containing F-RBP-Jκ, the use of the cell lysate containing both F-RBP-Jκ and F-ORF50(1–490) in EMSAs resulted in the formation of three extra supershifted complexes, probably corresponding to the F-RBP-Jκ/F-ORF50(1–490)/DNA complexes (Fig. 5E, left, red arrows). Under the same assay conditions, an EMSA was also performed using a previously identified ORF50 RE from the ORF47 promoter (designated ORF47p-RE) (27), which has a consensus RBP-Jκ-binding site (Fig. 5C and E). However, only a band corresponding to the F-RBP-Jκ/F-ORF50(1–490)/DNA complex was detected using the ORF47p-RE probe in the EMSA (Fig. 5E, right, red arrow). The differences between the two EMSA results raised the possibility that additional unknown protein components were involved in complex formation on the 56p-RE element. To find out the potential docking site for the unknown cellular protein(s) in the 56p-RE element, different mutant elements (including M1, M2, M3, and M4) shown in Fig. 5C were used as probes in EMSAs (Fig. 5F and G). The results from EMSAs showed that the 56p-RE(M1) element could not be bound by F-RBP-Jκ, F-ORF50(1–490), or the combination of F-RBP-Jκ and F-ORF50(1–490) (Fig. 5F). Although F-RBP-Jκ bound to the 56p-RE(M2), 56p-RE(M3), and 56p-RE(M4) elements, we noticed that only a predominant band corresponding to the F-RBP-Jκ/F-ORF50(1–490)/DNA complex was detected in EMSAs using the protein lysate containing F-RBP-Jκ and F-ORF50(1–490) (Fig. 5F and G, red arrows). Taken together, two major conclusions could be drawn from these results. First, the RBP-Jκ-binding site was located near the 5′ end of the 56p-RE element. Second, the 3′ region of the 56p-RE element was essential for supporting the formation of additional F-RBP-Jκ/F-ORF50(1–490)-associated complexes in EMSAs.

### Defining the RBP-Jκ-binding sequence in the ORF56 promoter.

**FIG 6:**
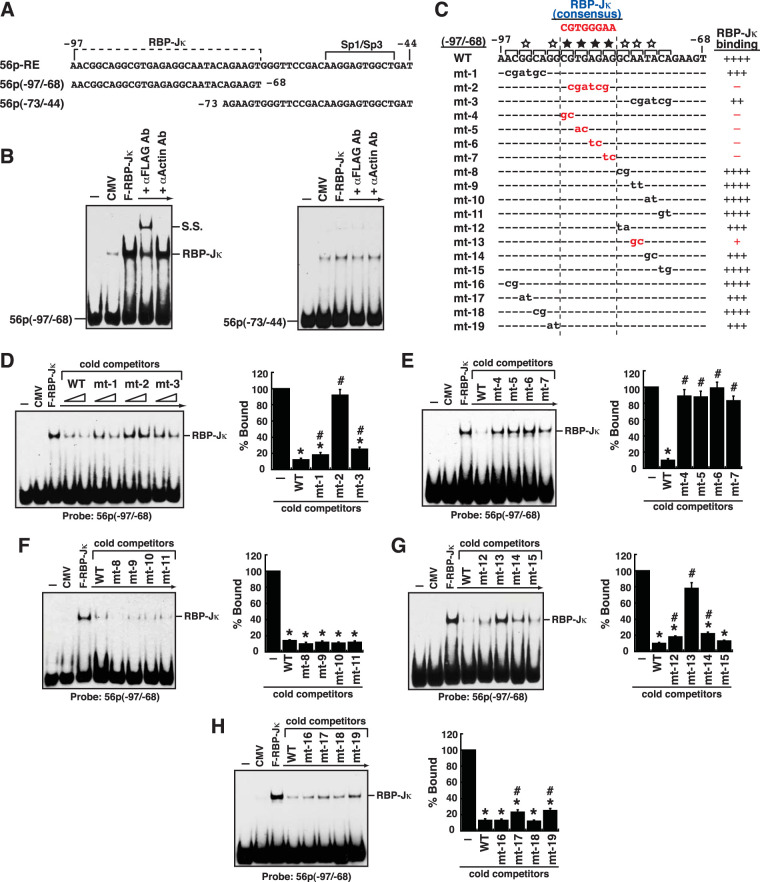
A nonconsensus RBP-Jκ-binding site is found in the 56p-RE element. (A) DNA sequences of the 56p-RE, 56p(−97/−68), and 56p(−73/−44) elements. (B) Specific binding of F-RBP-Jκ to the 56p(−97/−68) element but not to the 56p(−73/−44) element. EMSAs were performed using the protein lysate containing F-RBP-Jκ, and an anti-FLAG antibody was used for the supershift assay. (C) Summary of relative affinities of the wild-type (WT) and mutant 56p(−97/−68) elements for F-RBP-Jκ binding. Black stars indicate the nucleotide positions in the 56p(−97/−68) element essential for the binding of F-RBP-Jκ, whereas white stars indicate the auxiliary sequences for the binding of F-RBP-Jκ. The consensus RBP-Jκ-binding sequence is shown at the top of the sequence lines. (D to H) Binding competition analysis by EMSAs. EMSAs were performed using the protein extract containing F-RBP-Jκ. The indicated cold competitors shown in panel C were added at a concentration of a 30- or 50-fold molar excess relative to the labeled probe. In the right panels, the intensities of the F-RBP-Jκ/DNA complex in the gel were quantified by densitometry. *, *P < *0.05, for results compared to those with no competitor; #, *P < *0.05, for results compared to those with the wild-type 56p(−97/−68) competitor (*n *= 3).

To confirm our hypothesis that RBP-Jκ bound to the 5′ region of the 56p-RE element, two partially overlapping DNA fragments, 56p(−97/−68) and 56p(−73/−44), were used as probes in EMSAs (Fig. 6A). Our results clearly showed that the F-RBP-Jκ protein bound to the 56p(–97/–68) element but not the 56p(−73/−44) element (Fig. 6B). Since a consensus RBP-Jκ-binding sequence in the 56p(−97/−68) element could not be found, the RBP-Jκ-binding site was mapped by competition assays using a series of mutated 56p(−97/−68) motifs as cold competitors in EMSAs (Fig. 6C to H). Among them, the mutant motifs with point mutations at the internal region of the 56p(−97/−68) element, especially mt-2, mt-4, mt-5, mt-6, and mt-7, failed to compete for the binding of F-RBP-Jκ to the probe DNA in EMSAs (Fig. 6D and E), indicating that these mutated positions in the 56p(−97/−68) element were critical for F-RBP-Jκ binding. The identified core binding sequence of RBP-Jκ was 5′-CGTGAGAG, which is similar to a consensus sequence, 5′-CGTGGGAA (43). We additionally found that some specific nucleotides flanking the core RBP-Jκ-binding site in the 56p(−97/−68) element also influenced F-RBP-Jκ binding to a certain extent (Fig. 6C). For example, compared to the wild-type 56p(−97/−68) element as a cold competitor in EMSAs, the mutant elements, including mt-12, mt-13, mt-14, mt-17, and mt-19, showed reduced activity to compete for the binding of RBP-Jκ to the probe DNA (Fig. 6G and H).

### Cooperative association of ORF50, RBP-Jκ, and Sp3 on the 56p-RE element.

**FIG 7:**
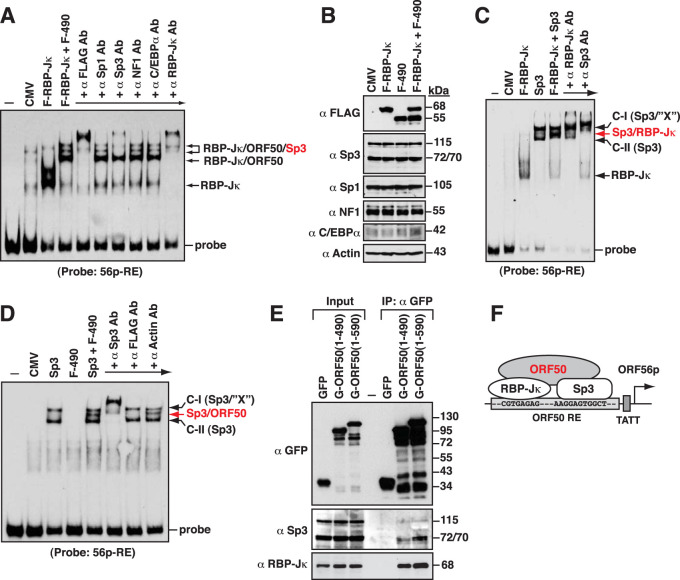
Sp3 is a key component of the protein complexes formed on the 56p-RE motif. (A) Antibody supershift or blockade assay by an EMSA using the 56p-RE probe. For the EMSA, the protein extracts from 293T cells transfected with the F-RBP-Jκ expression plasmid or both the F-RBP-Jκ and F-ORF50(1–490) expression plasmids were used, and specific antibodies against the FLAG tag, Sp1, Sp3, NF1, C/EBPα, and RBP-Jκ were included in the supershift/blocking test. The positions of the putative protein/DNA complexes are indicated. (B) Western blot analysis of Sp3, Sp1, NF1, and C/EBPα in 293T cells that were transfected with the plasmid expressing F-RBP-Jκ or F-ORF50(1–490) or both expression plasmids. (C) EMSA using the protein extracts containing F-RBP-Jκ, Sp3-F, or both F-RBP-Jκ and Sp3-F. When the protein lysate containing Sp3-F was used in the EMSA, two predominant Sp3/DNA complexes (designated C-I and C-II) were observed on the 56p-RE element. The C-I complex was proposed to be composed of Sp3-F and an unknown cellular protein, “X.” As noted, the Sp3/RBP-Jκ/DNA complex was formed between complexes C-I and C-II. (D) EMSA using the protein extracts of 293T cells that were transfected with a plasmid expressing untagged Sp3 or F-ORF50(1–490) or both expression plasmids. Antibodies specific to Sp3 or a FLAG tag were included in the supershift/blocking assay. (E) Coimmunoprecipitation analysis of the interaction between GFP-tagged ORF50 and Sp3 or RBP-Jκ in 293T cells. In the assay, 293T cells were transfected with the expression plasmid for GFP, GFP-ORF50(1–490) [G-ORF50(1–490)], or GFP-ORF50(1–590). Cell lysates were subjected to immunoprecipitation (IP) using anti-GFP antibody, and the immunoprecipitates were analyzed by Western blotting using anti-GFP, anti-Sp3, or anti-RBP-Jκ antibody. (F) Model for the full assembly of proteins, including RBP-Jκ, Sp3, and ORF50, on the 56p-RE element.

According to our EMSA results, the 5′ region of the 56p-RE element could be bound by RBP-Jκ (Fig. 5 and 6), whereas the 3′ region of the 56p-RE element could be bound by Sp-related proteins such as Sp1 or Sp3 (Fig. 4). Intriguingly, when the protein lysate containing F-RBP-Jκ and F-ORF50(1–490) was used in EMSAs, we found that a couple of uncharacterized protein complexes were additionally formed on the 56p-RE element (Fig. 5E and Fig. 7A). To characterize the protein component of these protein/DNA complexes on the 56p-RE element, specific antibodies against the FLAG tag, Sp1, Sp3, NF1, C/EBPα, and RBP-Jκ were individually added to EMSA reaction mixtures to remove or supershift the protein complexes. Our results showed that Sp3, but not Sp1, NF1, or C/EBPα, was predominantly present in the two slower-migrating F-RBP-Jκ/F-ORF50(1–490)-associated complexes (Fig. 7A, RBP-Jκ/ORF50/Sp3). Since both Sp1 and Sp3 often bind to the same cognate DNA elements with similar affinities, the recruitment of Sp3, but not Sp1, to the F-RBP-Jκ/F-ORF50(1–490)-associated complexes might be due to an increased Sp3/Sp1 ratio in the protein lysate of 293T cells that were transfected with the F-RBP-Jκ and F-ORF50(1–490) plasmids. However, Western blot analysis revealed that the overexpression of F-RBP-Jκ, F-ORF50(1–490), or both F-RBP-Jκ and F-ORF50(1–490) in 293T cells did not alter the expression levels of Sp1 and Sp3 (Fig. 7B). Notably, in addition to full-length Sp3 (115 kDa), two shorter Sp3 isoforms (72 and 70 kDa) arising from internal initiation of translation (47, 48) were also detected by Western blot analysis (Fig. 7B). To further investigate the binding of Sp3 to the 56p-RE element, the protein lysates of 293T cells transfected with the expression plasmid encoding F-RBP-Jκ, FLAG-tagged Sp3 (Sp3-F), or both expression plasmids were used in EMSAs. We found that a prominent F-RBP-Jκ/DNA complex was detected in EMSAs using the protein lysate containing F-RBP-Jκ, whereas two major protein complexes, a slower-migrating (C-I) and a faster-migrating (C-II) complex, were detected using the protein lysate containing Sp3-F (Fig. 7C). When the protein lysate containing both Sp3-F and F-RBP-Jκ was used in EMSAs, an additional protein complex that migrated to a position between the C-I and C-II complexes was observed, probably corresponding to the Sp3/RBP-Jκ/DNA complex (Fig. 7C, Sp3/RBP-Jκ). In the antibody supershifting/blocking test, we found that the putative Sp3/RBP-Jκ/DNA complex could be specifically removed by anti-RBP-Jκ or anti-Sp3 antibody in EMSAs (Fig. 7C, Sp3/RBP-Jκ). Here, we noticed that only the C-II complex, but not the C-I complex, allowed the recruitment of F-RBP-Jκ (Fig. 7C, Sp3/RBP-Jκ). In this case, the C-I complex might be composed of Sp3-F and an unknown protein (Fig. 7C, Sp3/“X”).

On the other hand, the protein lysates of 293T cells transfected with the plasmid expressing untagged Sp3 or F-ORF50(1–490) or both expression plasmids were also used in EMSAs. As expected, F-ORF50(1–490) in the prepared cell lysate was not sufficient to bind the 56p-RE element in EMSAs, whereas there were two Sp3/DNA complexes, C-I and C-II, detected in EMSAs using the cell lysate containing overexpressed Sp3 (Fig. 7D). In particular, when the protein lysate containing both overexpressed Sp3 and F-ORF50(1–490) was used in EMSAs, an extra protein/DNA complex located between the C-I and C-II complexes could be detected (Fig. 7D). This extra protein/DNA complex appeared to be an Sp3/F-ORF50(1–490)/DNA complex (Fig. 7D, Sp3/ORF50) because it could be specifically supershifted or removed by adding anti-Sp3 or anti-FLAG antibody in EMSAs. The formation of the Sp3/F-ORF50(1–490)/DNA complex strongly implied that the ORF50 protein might interact with Sp3. To demonstrate the interaction between ORF50 and Sp3, we performed coimmunoprecipitation experiments using the protein lysates from 293T cells that were transfected with a plasmid expressing green fluorescent protein (GFP), GFP-ORF50(1–490), or GFP-ORF50(1–590) (Fig. 7E). After immunoprecipitation using anti-GFP antibody, we found that Sp3 could be coimmunoprecipitated with GFP-ORF50(1–490) or GFP-ORF50(1–590) but not GFP (Fig. 7E). In the same immunoprecipitates, we additionally found that RBP-Jκ was also coimmunoprecipitated with GFP-ORF50(1–490) or GFP-ORF50(1–590) but not GFP (Fig. 7E). Based on these results, a fully active transcriptional complex formed on the ORF56 promoter could include RBP-Jκ, ORF50, and Sp3 together (Fig. 7F).

### Synergistic activation of the 56p-RE-containing reporter construct by ORF50 and Sp3.

**FIG 8:**
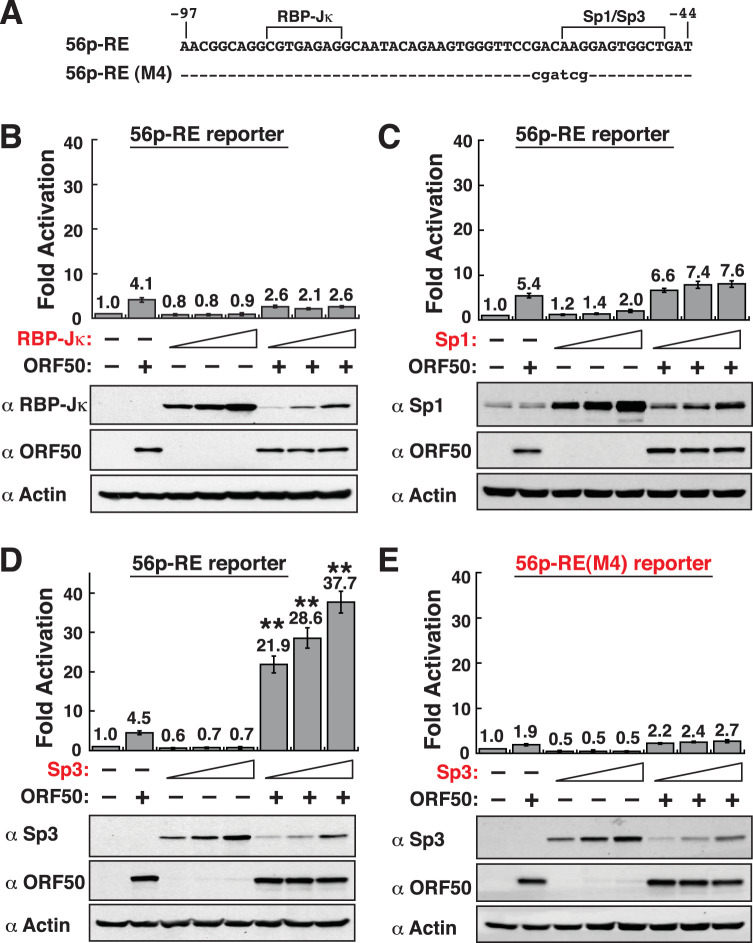
Sp3 acts in synergy with ORF50 to activate the 56p-RE-containing reporter construct. (A) DNA sequences of the wild-type and mutant 56p-RE elements. The 56p-RE(M4) element harbors point mutations at the Sp1/Sp3-binding site. (B) Effect of increasing amounts of RBP-Jκ alone or together with ORF50 on the activation of the 56p-RE reporter construct. (C) Effect of increasing amounts of Sp1 alone or together with ORF50 on the activation of the 56p-RE reporter construct. (D) Effect of increasing amounts of Sp3 alone or together with ORF50 on the activation of the 56p-RE reporter construct. **, synergistic activation by ORF50 and Sp3. (E) Effect of increasing amounts of Sp3 alone or together with ORF50 on the activation of the 56p-RE(M4) reporter construct. For all transfection experiments, a fixed amount (600 ng) of plasmid DNA was transfected into HKB5/B5 cells, and reporter assays were performed 48 h after transfection. The transfected DNA constructs included the reporter plasmid (100 ng), the ORF50 expression plasmid (100 ng), and increasing amounts (100, 200, and 400 ng) of the RBP-Jκ, Sp1, or Sp3 expression plasmid. In each transfection, the empty vector pCMV was added to keep the total amount of DNA constant. Western blot analyses were performed to detect the expression of transfected proteins, including RBP-Jκ, Sp1, Sp3, or ORF50, in HKB5/B5 cells.

As described above, the 56p-RE element could be bound by RBP-Jκ, Sp1, or Sp3. We next investigated whether cotransfection of the F-RBP-Jκ, Sp1, or Sp3-F expression plasmid into HKB5/B5 cells could further enhance the ORF50-mediated activation of the 56p-RE reporter construct (Fig. 8). In the control group, cotransfection of increasing amounts of the F-RBP-Jκ, Sp1, or Sp3-F expression plasmid alone with the 56p-RE reporter construct did not substantially enhance the luciferase activity (Fig. 8B to D). Cotransfection of increasing amounts of the F-RBP-Jκ or Sp1 expression plasmid also did not further increase the ORF50-mediated reporter activation of the 56p-RE construct (Fig. 8B and C). However, the ectopic expression of Sp3-F was able to cooperate with ORF50 to synergistically activate the reporter activity of the 56p-RE construct (Fig. 8D). Noteworthily, Western blot analysis revealed that the synergistic activation of the 56p-RE reporter construct by Sp3-F and ORF50 was not due to a significant upregulation of ORF50 in transfected cells (Fig. 8D). To further examine whether the binding of Sp3 to the 56p-RE element was essential for synergy, the 56p-RE(M4) reporter construct that harbors point mutations at the Sp1/Sp3-binding site was included in reporter assays (Fig. 8A and E). As shown in Fig. 8E, the coexpression of ORF50 and Sp3-F did not cause synergistic activation of the 56p-RE(M4) reporter construct. Our results therefore suggested that ORF50 and Sp3 act in synergy to activate the ORF56 promoter, mainly through the Sp1/Sp3-binding motif.

### Synergistic activation of viral or cellular promoters by ORF50 and Sp3.

**FIG 9:**
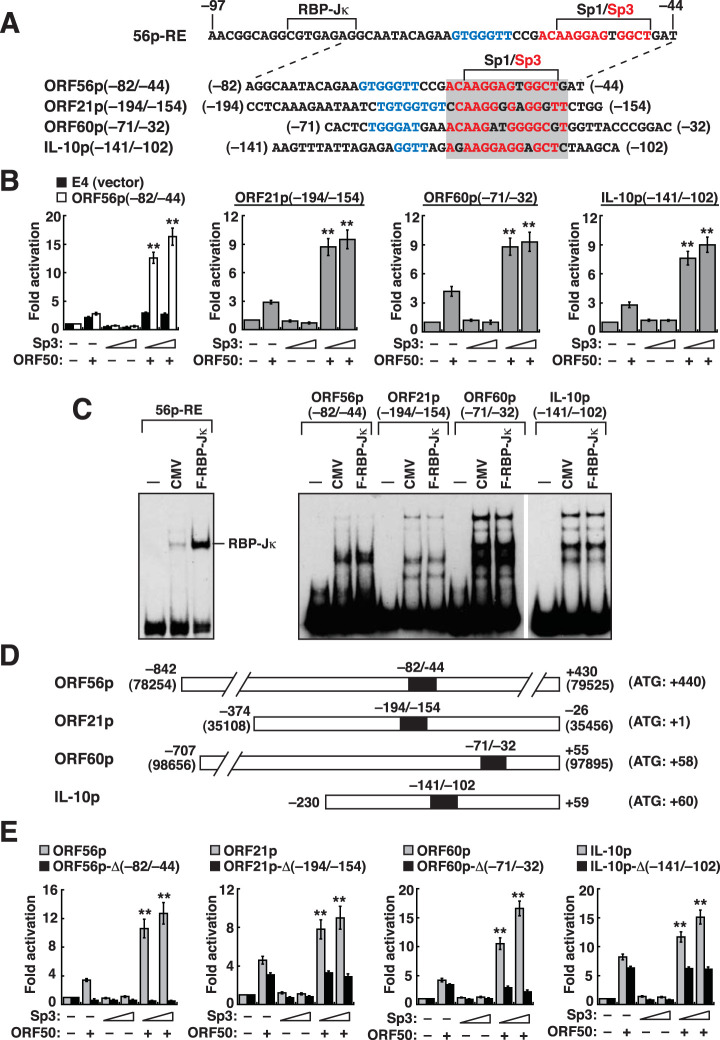
ORF50 cooperates with Sp3 to synergistically activate the ORF21, ORF60, and IL-10 promoters. (A) Sequence alignment of the ORF56p(−82/−44), ORF21p(−194/−154), ORF60p(−71/−32), and IL-10p(−141/−102) elements. All elements share substantial sequence homology at the Sp1/Sp3-binding site (gray box). Upstream of the Sp1/Sp3-binding site, a GT-rich sequence motif was found in these elements (blue font). (B) Transcriptional activation of the reporter constructs containing the indicated promoter elements by ORF50 and Sp3. HKB5/B5 cells were transfected with a fixed amount (600 ng) of plasmid DNA, which included the indicated reporter construct (100 ng), the ORF50 expression plasmid (100 ng), and the Sp3 expression plasmid (200 or 400 ng). The empty vector pCMV was added to keep the total amount of DNA equivalent for each transfection. Forty-eight hours after transfection, cells were harvested, and reporter assays were carried out. **, synergistic activation by ORF50 and Sp3. (C) EMSAs using the 56p-RE, ORF56p(−82/−44), ORF21p(−194/−154), ORF60p(−71/−32), and IL-10p(−141/−102) elements as probes. The protein lysate containing F-RBP-Jκ was used in EMSAs. (D) Diagram of the constructed ORF56, ORF21, ORF60, and IL-10 natural promoters. The black box in each promoter indicates the location of the identified conserved ORF50/Sp3-responsive motif. Numbers in parentheses are the nucleotide positions of the KSHV genome (GenBank accession no. NC_009333). (E) Transcriptional activation of reporter constructs containing the indicated natural promoters and their mutants by ORF50 and Sp3. The ORF56p-Δ(−82/−44), ORF21p-Δ(−194/−154), ORF60p-Δ(−71/−32), and IL-10-Δ(−141/−102) constructs are internal deletion mutants that lack the conserved ORF50/Sp3-responsive motif. **, synergistic activation by ORF50 and Sp3.

Since the 56p-RE element contains both RBP-Jκ- and Sp3-binding sites, we next determined whether the RBP-Jκ-binding sequence in the 56p-RE element played a role in transcriptional synergy mediated by ORF50 and Sp3. The 56p(−82/−44)-containing reporter construct that lacks the RBP-Jκ-binding site in the 56p-RE element was included in the assay of synergy by ORF50 and Sp3 (Fig. 9A). Compared to the control construct pE4-luc, the 56p(−82/−44) reporter construct still produced a strong synergistic response to ORF50 and Sp3 (Fig. 9A and B), indicating that the RBP-Jκ-binding site in the 56p-RE element was dispensable for the synergy by ORF50 and Sp3. After sequence similarity searches, a number of DNA elements similar to the 56p(−82/−44) element were found. Among these predicted elements, three promoter motifs were selected for further investigation, viral ORF21p from positions −194 to −154 (relative to the translation start site), viral ORF60p from positions −71 to −32 (relative to the transcriptional start site), and cellular IL-10p from positions −141 to −102 (relative to the transcriptional start site) (Fig. 9A). These promoter elements share a similar Sp1/Sp3-binding site and a GT-rich sequence flanking the Sp1/Sp3-binding site (Fig. 9A). When the reporter construct containing the ORF21p(−194/−154), ORF60p(−71/−32), or IL-10p(−141/−102) element was individually cotransfected with the ORF50 expression plasmid alone into HKB5/B5 cells, these elements conferred only weak ORF50 responsiveness. However, cotransfection of the ORF50 and Sp3-F expression plasmids substantially promoted synergistic activation of the reporter constructs containing the ORF21p(−194/−154), ORF60p(−71/−32), or IL-10p(−141/−102) element (Fig. 9B). It should be noted that these selected DNA elements did not contain a functional RBP-Jκ-binding site, as evaluated by EMSAs using the cell lysate containing F-RBP-Jκ (Fig. 9C). To further examine whether ORF50 and Sp3 synergistically activated the natural promoters, promoter fragments of the ORF21, ORF60, and IL-10 genes, including ORF21p(−374/−26), ORF60p(−707/+55), and IL-10p(−230/+59), were individually cloned into pGL3-Basic (Fig. 9D). Concurrently, we sought to determine whether the identified ORF50/Sp3-responsive elements were essential for mediating transcriptional synergy in these natural promoters. Specific internal deletions of the corresponding ORF50/Sp3-responsive elements in the ORF56p-FL, ORF21p(−374/−26), ORF60p(−707/+55), and IL-10p(−230/+59) promoters were also generated (Fig. 9D). The resultant reporter constructs were then individually cotransfected with the ORF50 expression plasmid, the Sp3-F expression plasmid, or both expression plasmids into HKB5/B5 cells. As shown in Fig. 9E, we consistently found that the ORF56p-FL, ORF21p(−374/−26), ORF60p(−707/+55), and IL-10p(−230/+59) reporter constructs could be notably activated by the combination of ORF50 and Sp3-F. However, the internal deletion mutant constructs, including the ORF56p-Δ(−82/−44), ORF21p-Δ(−194/−154), ORF60p-Δ(−71/−32), and IL-10p-Δ(−141/−102) constructs, which lack the ORF50/Sp3-responsive element in the natural promoters, completely lost the synergistic response to ORF50 and Sp3 (Fig. 9E).

### Specific DNA sequence requirements for transcriptional synergy by ORF50 and Sp3.

**FIG 10:**
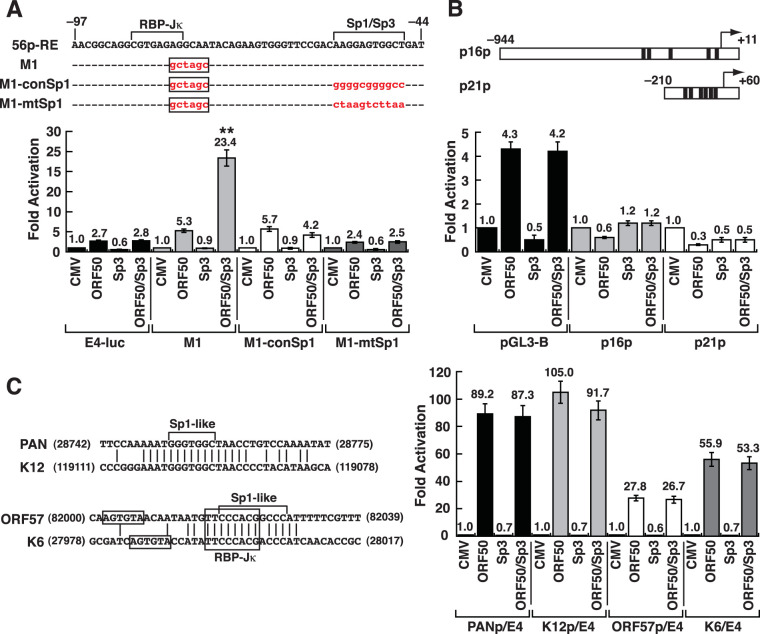
The consensus high-affinity Sp1/Sp3-binding sequences or the ORF50 REs from the PAN, K12, ORF57, and K6 promoters do not confer a synergistic response to ORF50 plus Sp3. (A) Transcriptional activation of the 56p-RE(M1)-containing reporter construct and its mutants by ORF50 and Sp3. Two 56p-RE(M1) mutant constructs that converted the nonconsensus Sp1/Sp3-binding sequence in the 56p-RE(M1) element to a consensus high-affinity Sp1-binding site (GGGGCGGGGCC) (conSp1) or to a nonfunctional Sp1-binding site (CTAAGTCTTAA) (mtSp1) are shown at the top. As noted, the 56p-RE(M1) element carries an inactivating RBP-Jκ-binding site. **, synergistic activation by ORF50 and Sp3. (B) Effect of ORF50 and Sp3 on the transcription of the p16 and p21 gene promoters. The p16 and p21 promoters contain five and six consensus Sp-binding sites (black boxes), respectively. A luciferase reporter assay was performed 48 h after transfection. (C) Transcriptional activation of the reporter constructs containing ORF50 REs from the PAN, K12, ORF57, and vMIP-1 promoters by ORF50 and Sp3. All these ORF50 REs contain a predicted Sp1/Sp3-binding site (left). The numbers in the diagram are the nucleotide positions of the KSHV genome (GenBank accession no. NC_009333). A luciferase reporter assay was performed in HKB5/B5 cells that were transfected with the indicated reporter constructs and effectors for 48 h.

Due to the importance of the Sp1/Sp3-binding site in the 56p-RE element for transcriptional synergy, we next addressed whether other Sp-related binding motifs were able to confer a synergistic response to ORF50 and Sp3. To test this, the Sp1/Sp3-binding sequence (5′-AAGGAGTGGCT) was converted to a consensus high-affinity Sp1/Sp3-binding sequence (5′-GGGGCGGGGCC), or to a nonfunctional Sp1/Sp3-binding sequence (5′-CTAAGTCTTAA), in the 56p-RE(M1) element that carries an inactivating RBP-Jκ-binding site (Fig. 5 and Fig. 10A). These two mutant reporter constructs were designated 56p-RE(M1)-conSp1 and 56p-RE(M1)-mtSp1, respectively (Fig. 10A). In the transactivation assay, we found that the 56p-RE(M1) reporter construct was still sufficient to conduct a synergistic response to ORF50 and Sp3-F; however, the transcriptional synergy was not seen in either the 56p-RE(M1)-conSp1 or 56p-RE(M1)-mutSp1 reporter construct (Fig. 10A). To further confirm our results, two cellular promoters, the p16 and p21 gene promoters, which contain five and six consensus Sp-binding sites, respectively, were chosen for testing their transcriptional response to ORF50 and Sp3 (Fig. 10B). The tandem Sp1/Sp3-binding sites in the p16 and p21 promoters are known to play critical roles in their basal promoter activity (51, 52). Consistently, transcriptional synergy could not be detected for the p16 and p21 promoters (Fig. 10B). These results highlighted that the consensus high-affinity Sp1/Sp3-binding sites do not necessarily confer active Sp3/ORF50 recruitment and synergy.

There are two major subclasses of ORF50 REs previously characterized in other lytic gene promoters of KSHV. The ORF50 REs from the PAN and K12 promoters are the best-known direct targets of ORF50 (24, 36), whereas the ORF50 REs from the ORF57 and K6 promoters are typical examples of indirect ORF50 targets with a consensus RBP-Jκ-binding site (26) (Fig. 10C). However, based on the nucleotide sequence, the newly identified ORF50 REs from the ORF56, ORF21, ORF60, and IL-10 promoters appeared to be different from the above-mentioned subclasses. Despite differences in the nucleotide sequences, each of the ORF50 REs from the PAN, K12, ORF57, and K6 promoters contains a putative Sp1/Sp3-binding site (Fig. 10C). We therefore determined whether Sp3 could act in synergy with ORF50 to activate these ORF50 REs from the PAN, K12, ORF57, and K6 promoters. Nevertheless, the coexpression of Sp3-F did not further enhance the ORF50 responsiveness of these reporter constructs (Fig. 10C). Together, our results suggested that the newly identified ORF50 REs from the ORF56, ORF21, ORF60, and IL-10 promoters could be classified as a new subclass of ORF50 REs.

## DISCUSSION

In this report, we demonstrate that the ORF56 gene promoter is a bona fide transcriptional target of ORF50 and show that Sp3 can cooperate with ORF50 to synergistically activate the gene promoter through a newly identified ORF50 RE located between positions −97 and −44 (designated 56p-RE). Although the 56p-RE element includes both the RBP-Jκ- and Sp1/Sp3-binding sites, ORF50/Sp3-mediated transcriptional synergy is mainly dependent on the Sp1/Sp3-binding site. Furthermore, we find that the promoters of the viral ORF21 and ORF60 genes as well as the cellular IL-10 gene contain a sequence motif similar to the 56p-RE element of the ORF56 promoter, and these promoters can also be synergistically activated by ORF50 and Sp3. Understanding the synergistic effect of ORF50 and Sp3 on the transcription of specific viral and cellular genes may be important not only for providing new information about ORF50-mediated transactivation but also for giving further insights into the role of Sp3 in KSHV lytic reactivation.

### Identification of the ORF56 promoter as a bona fide target of the ORF50 protein.

Although ORF50 is considered able to transactivate most of the KSHV early lytic genes (20), the molecular mechanism involved in the transcriptional activation of a small subset of early lytic genes, particularly the genes encoding enzymes necessary for viral DNA replication, by ORF50 remains largely unknown. Examples of such genes include the viral ORF56 (primase), ORF9 (DNA polymerase), ORF44 (helicase), and ORF40/41 (primase-associated factor) genes. Part of the reason for the lack of literature addressing this issue may be because these DNA replication enzymes are generally expressed at very low levels, and their promoters tend to be activated weakly when stimulating signals arrive. In the present study, we found that the ORF56 promoter possesses two unique features that constrain promoter activity and its ORF50 responsiveness in certain cell types (e.g., HKB5/B5 cells). One is that the ORF56 promoter contains a noncanonical TATA box motif (TATT), and the other is that the identified 54-bp ORF50 RE in the promoter confers only weak ORF50 responsiveness under normal inductive conditions. The TATT box motif is rarely found in the core promoters of KSHV early lytic genes but is frequently present in those of true late genes (53, 54). This observation implicated that the ORF56 promoter could be transcriptionally activated at both the early and early-late stages during the lytic cycle. Additionally, the TATT motif in the ORF56 promoter apparently affected the overall response to ORF50 in KSHV-negative cell lines such as HKB5/B5 (Fig. 1Eiii). The conversion of the TATT motif in the ORF56 promoter to a consensus TATA box markedly improves ORF50 responsiveness (Fig. 1F). As for the newly identified ORF50 RE from the ORF56 promoter (56p-RE), it contains a nonconsensus RBP-Jκ-binding sequence, 5′-CGTGAGAG, which is somewhat different from the consensus sequence 5′-CGTGGGAA (43). EMSAs also showed that specific nucleotides flanking the nonconsensus RBP-Jκ-binding sequence were also required for the full binding of RBP-Jκ (Fig. 6C to H). Since the RBP-Jκ/DNA complex was not detected in EMSAs using either untreated or SB-treated HH-B2 cell lysates (Fig. 4 and data not shown), the identified RBP-Jκ-binding site in the 56p-RE element seems to be a weak binding site for RBP-Jκ compared to those present in other known ORF50 REs (e.g., ORF57p-RE, K6p-RE, ORF47p-RE, and ORF45p-RE) (26–28). Consistent with this notion, the RBP-Jκ-binding portion of the 56p-RE element did not confer noticeable ORF50 responsiveness (Fig. 2, RE-7 and RE-8). In addition to the nonconsensus RBP-Jκ-binding site, the 56p-RE element also has a nonconsensus Sp1/Sp3-binding site. The Sp1/Sp3-binding portion itself was also not enough to confer substantial ORF50 responsiveness under normal inductive conditions. Despite a weak response to ORF50 alone, the 56p-RE-containing reporter construct was greatly activated by the combination of ORF50 and Sp3. Our data collectively support that the ORF56 gene promoter is a transcriptional target of ORF50; however, efficient transcriptional activation of the promoter by ORF50 requires cooperation with Sp3.

### Synergistic activation of viral and cellular promoters by Sp3 and ORF50.

**FIG 11:**
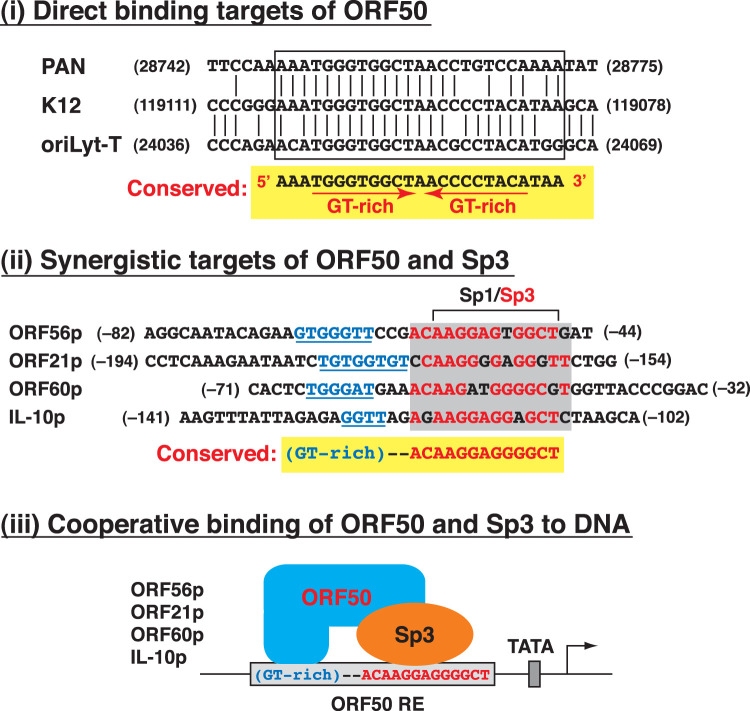
Sequence comparison of direct ORF50-binding targets and the newly identified ORF50/Sp3-responsive targets. (i) Direct ORF50-binding elements. The ORF50 REs from the PAN, K12, and oriLyt-T genes share a conserved core element that consists of two GT-rich (imperfect) inverted repeats. The numbers in the diagram represent the nucleotide positions of the KSHV genome (GenBank accession no. NC_009333). (ii) ORF50/Sp3-responsive elements. The ORF50 REs from the ORF56, ORF21, ORF60, and IL-10 promoters share a nonconsensus Sp1/Sp3-binding motif and a GT-rich region flanking the Sp1/Sp3-binding motif. (iii) Model for the cooperative binding of ORF50 and Sp3 to the conserved *cis*-acting motif present in the ORF56, ORF21, ORF60, and IL-10 promoters. The GT-rich region in the identified ORF50 REs may function as a docking site for ORF50 to stabilize the ORF50/Sp3 complex on the promoter DNA.

Based on the findings of the present study, here, we propose that the activation of the ORF56 promoter by ORF50 may involve at least two different mechanisms, including the recruitment of the ORF50/RBP-Jκ or ORF50/Sp3 complex to the 56p-RE element (Fig. 7F). Particularly, we found that Sp3, but not RBP-Jκ, cooperates with ORF50 to synergistically activate the 56p-RE-containing reporter construct (Fig. 8). Although the RBP-Jκ-binding sequence of the 56p-RE element is required for a full response to ORF50, deleting or inactivating the RBP-Jκ-binding site of the 56p-RE element, such as 56p(−82/−44) or 56p-RE(M1), still produces a strong synergistic response to ORF50 and Sp3 (Fig. 9B and Fig. 10A). All our experiments highlighted that ORF50/Sp3-mediated transcriptional synergy is mainly dependent on the Sp1/Sp3-binding motif of the 56p-RE element. However, it is worth noting that the high-affinity Sp-binding motifs do not necessarily reflect a strong synergistic response to ORF50 and Sp3 (Fig. 10A and B). In addition to the nonconsensus Sp1/Sp3-binding site, a GT-rich motif (GTGGGTT) immediately flanking the Sp1/Sp3-binding site was noticed in the 56p-RE element. The presence of the GT-rich motif in the identified 56p-RE element is reminiscent of the ORF50 REs from the PAN, K12, and oriLyt-T gene promoters (24, 25). These ORF50 REs share a highly conserved nucleotide sequence, AAATGGGTGGCTAACCCCTACATAA, where two imperfect GT-rich inverted repeats, 5′-TGGGTGGCT and 5′-ACCCCTACA, could be found (Fig. 11i). Although direct binding of the full-length ORF50 or its DNA-binding domain to the 56p-RE element was not detected in EMSAs (Fig. 4B and Fig. 5E), it is possible that the GT-rich motif could serve as a potential docking site for ORF50 to stabilize the ORF50/Sp3 complex on the promoter DNA (Fig. 11ii and iii), thereby promoting transcriptional synergy.

After sequence similarity searches, up to 30 sequence motifs similar to the 56p-RE element could be found in the viral or cellular genome; however, only a small proportion of them were located in known promoter regions. Here, we selected three promoter elements for further characterization, the ORF21p(−194/−154), ORF60p(−71/−32), and IL-10p(−141/−102) elements (Fig. 9). These promoter elements share a conserved Sp1/Sp3-binding motif (5′-ACAAGGAGGGGCT) and a flanking GT-rich region (Fig. 11ii). Our data indeed showed that the ORF21, ORF60, and IL-10 gene promoters could be synergistically activated by ORF50 and Sp3 (Fig. 9). In agreement with previous studies, both the ORF21 and ORF60 promoters are already known to be transcriptional targets of ORF50, and the conserved Sp1/Sp3-binding motifs identified in this study are specifically located within their ORF50 REs (30, 37). IL-10 is an immunosuppressive cytokine that may act as a key factor contributing to viral persistence by suppressing host immunity (55). Previous studies have shown that high levels of IL-10 could be detected in KSHV-associated MCD (KSHV-MCD), which had a higher proportion of lytic cells than KS or PELs (56). Moreover, Caro-Vegas et al. (57) found that the production of IL-10 in a patient with KSHV-associated inflammatory cytokine syndrome (KICS) was positively correlated with KSHV loads during disease progression (57). All these studies implied that the upregulation of IL-10 is closely associated with KSHV lytic reactivation. Recently, Miyazawa et al. (58) also reported that the ORF50 protein was able to activate the IL-10 promoter via interactions with Sp1 and Sp3. In our study, we found an ORF50 RE carrying an Sp1/Sp3-binding site in the IL-10 promoter (Fig. 9). Taken together, the newly identified ORF50/Sp3-responsive elements, which contain a nonconsensus Sp1/Sp3-binding motif and a flanking GT-rich region, from the ORF56, ORF21, ORF60, and IL-10 promoters could be grouped as a new subclass of ORF50 REs.

### Potential regulation of Sp3 in the KSHV lytic cycle.

Sp3 is a ubiquitously expressed transcription factor that regulates a wide variety of genes involved in multiple cellular processes (47). Due to the structural similarities in the C-terminal DNA-binding domains, Sp3 and other Sp/KLF family members may have overlapping sets of target genes but may exhibit diverse effects on target gene regulation. In this study, although both Sp1 and Sp3 were able to bind to the 56p-RE element in EMSAs (Fig. 4), only Sp3 was recruited to the ORF50-associated complexes on the 56p-RE element (Fig. 7A). Consistently, Sp3, but not Sp1, acts in synergy with ORF50 to activate the ORF56 promoter (Fig. 8). Under normal conditions, endogenous Sp3 seems to be a limiting factor for the recruitment of a functional ORF50/Sp3 complex to target DNA in cells. However, the overexpression of Sp3 may allow the efficient formation of the ORF50/Sp3 complex on target DNA, thereby promoting the synergistic activation of target gene promoters. Currently, there are 27 Sp/KLF family members identified in humans, 9 members of the Sp subfamily (Sp1 to Sp9) and 18 members of the KLF subfamily (KLF1 to KLF18) (59, 60). It may be possible that these endogenous Sp/KLF members, such as Sp1, in cells could have the potential to negatively modulate the access of the ORF50/Sp3 complex to target DNA under certain conditions. A better understanding of the functional relationship between Sp3 and other Sp/KLF family members for transcriptional synergy with ORF50 may be an important direction for future research.

In summary, we demonstrate that ORF50 can cooperate with Sp3 to synergistically activate the viral ORF56, ORF21, and ORF60 gene promoters as well as the cellular IL-10 gene promoter through a conserved Sp1/Sp3-binding region. Our findings may help provide new insights into the complicated mechanisms of ORF50-mediated transactivation.

## MATERIALS AND METHODS

### Cell cultures, reagents, and transfections.

HH-B2 and BC3 are KSHV-infected PEL cell lines, which were grown in RPMI 1640 medium supplemented with 15% fetal bovine serum (FBS). For viral lytic induction, HH-B2 and BC3 cells were treated with 3 mM sodium butyrate. HKB5/B5, a cell clone formed by the fusion of HH514-16 cells with 293T cells (24), was cultured in RPMI 1640 medium supplemented with 8% FBS. The mouse embryonic fibroblast cell line OT13 (catalog no. RCB1927; RIKEN) and an RBP-Jκ^−/−^ cell line, OT11 (catalog no. RCB1926; RIKEN), were provided by the RIKEN BioResource Research Center through the National Bio-Resource Project of the MEXT, Japan (61). OT13 and OT11 cells were grown in high-glucose Dulbecco’s modified Eagle’s medium (DMEM) supplemented with 10% FBS and 100 U/ml of mouse interferon. 293T is a human embryonic kidney cell line transformed with the E1 region of adenovirus and the simian virus 40 T antigen. 293T cells were cultured in high-glucose DMEM supplemented with 10% FBS. For all transfection experiments, Lipofectamine 2000 or Lipofectamine 3000 transfection reagent (catalog no. 11668019 or L3000008; Invitrogen) was used according to the manufacturer’s recommendations.

### Rapid amplification of 5′ cDNA ends.

Details of the amplification of 5′ cDNA ends of the viral lytic transcripts using the GeneRacer kit (catalog no. L150202; Invitrogen) were described previously (30). Briefly, HH-B2 cells were treated with 3 mM sodium butyrate for 24 h, and total RNAs from the treated cells were prepared with the RNeasy minikit (catalog no. 74104; Qiagen). Two micrograms of total RNA was used for incubation with calf intestinal phosphatase to remove the 5′ phosphates of truncated mRNA and non-mRNA. After the decapping of full-length mRNAs by tobacco acid pyrophosphatase, an RNA linker oligonucleotide was ligated to mRNAs by using T4 RNA ligase. The first-strand cDNA was obtained by reverse transcription using random primers. Subsequently, samples were subjected to amplification by primary PCR and nested PCR using gene-specific primers and linker primers provided in the kit. The ORF56-specific primers used for primary PCR and nested PCR were 5′-ATCTCCAACAGCGGCTTGAACAACC and 5′-CAACACGGGCGAACACCTTGTGCCG, respectively. The amplified DNA fragments were cloned and sequenced. Eight of 13 RACE clones were initiated at nt 79096, 3 clones were initiated at nt 79092, 1 clone was initiated at nt 79131, and 1 clone was initiated at nt 79174.

### Plasmid construction.

Plasmids, including pCMV-FLAG-ORF50, pCMV-FLAG-ORF50(1–490), pCMV-FLAG-RBP-Jκ, pCMV-FLAG-Sp1(300–785), pCMV-GFP-ORF50(1–590), pCMV-GFP-ORF50(1–490), and pE4-luc, were described previously (21, 23, 26, 30). The ORF56 promoter from positions −842 to +430, corresponding to nt 78254 to 79525 of the KSHV genome (GenBank accession no. NC_009333), was amplified by PCR using total DNA of HH-B2 cells as a template and cloned into pGL3-Basic (Promega). Different deletions of the ORF56 promoter in pGL3-Basic were then generated. Specific point mutations in the ORF56 promoter were created using the QuikChange site-directed mutagenesis kit (Stratagene). Series of ORF50 response regions or elements in pE4-luc, a luciferase reporter containing an adenovirus E4 minimal promoter, were generated using PCR-amplified DNA fragments or double-stranded annealed oligonucleotides (24, 26). The pCMV-Sp3-FLAG plasmid that expresses Sp3 with a C-terminal FLAG tag was purchased from GenScript (product identification OHu28260D; GenBank accession no. NM_003111.4). The pCMV-Sp3 plasmid that encodes untagged Sp3 was constructed by inserting full-length Sp3 cDNA into pCMV-2 (Sigma-Aldrich). Similar to the generation of the ORF56p reporter construct, the ORF21 promoter region from positions −374 to −26, the ORF60 promoter region from positions −707 to +55, and the IL-10 promoter region from −230 to +59 were also cloned into pGL3-Basic. Furthermore, inverse PCR was applied to generate an internal deletion in the ORF56, ORF21, ORF60, or IL-10 promoter. The reporter constructs that contain the p16 or p21 promoter were gifts from Li-Kwan Chang (National Taiwan University, Taiwan).

### Luciferase assays.

For transient transfections, HH-B2, BC3, and HKB5/B5 cells were seeded at a density of 7 × 10^5^ cells/well in 24-well plates, whereas OT11 and OT13 cells were seeded at a density of 1.5 × 10^5^ cells/well in 24-well plates 1 day prior to transfection. All cell lines were transfected with a fixed amount (600 ng) of plasmid DNA that included the reporter and effector plasmids. Cells were collected 48 h after transfection, and the reporter assays were performed according to the manufacturer’s protocol for the luciferase reporter assay system (catalog no. E1501; Promega). Fold activation was calculated as the luciferase activity of the reporter construct in the presence of stimuli divided by that in the absence of stimuli. All the experiments were repeated at least three times with duplicate samples.

### Electrophoretic mobility shift assays.

Total protein extracts for electrophoretic mobility shift assays (EMSAs) were prepared from HH-B2 cells that were untreated or treated with SB (3 mM) for 24 h or from 293T cells that were transfected with the indicated expression plasmids for 24 h. These cells were lysed in a buffer containing 0.42 M NaCl, 20 mM HEPES (pH 7.5), 25% glycerol, 1.5 mM MgCl_2_, 0.2 mM EDTA, 1 mM dithiothreitol, 1 mM phenylmethylsulfonyl fluoride, and 2 μg aprotinin per ml. The DNA probes used in EMSAs were labeled with biotin-11-UTP using terminal deoxynucleotidyltransferase (catalog no. 89818; Pierce). In each EMSA reaction mixture, 12 μg of protein extracts was added to a buffer containing 10 mM HEPES (pH 7.5), 50 mM NaCl, 2 mM MgCl_2_, 2.5 μM ZnSO_4_, 0.5 mM EDTA, 1 mM dithiothreitol, 15% glycerol, and 0.3 μg poly(dI-dC) in a total volume of 20 μl. For competition assays, unlabeled competitor DNA was added to the initial reaction mix. Antibodies to Sp1 (catalog no. CS200631; Millipore), Sp3 (catalog no. sc-365220; Santa Cruz), actin (catalog no. sc-47778; Santa Cruz), the FLAG tag (catalog no. SI-A2220; Sigma), NF1 (catalog no. sc-5567; Santa Cruz), C/EBPα (catalog no. sc-9314; Santa Cruz), and RBP-Jκ (catalog no. ab25949; Abcam) were added to EMSA reaction mixtures for the supershift/blockade test. To confirm reproducibility, some specific experiments were repeated at least three times, and the intensities of specific protein/DNA complexes formed in EMSAs were quantified by densitometry using Quantity One software (Bio-Rad).

### Western blot analysis.

Western blot analysis was carried out as described previously (62). Briefly, cell protein extracts were resolved on an 8% to 10% polyacrylamide gel. After gel electrophoresis, the proteins were transferred onto polyvinylidene difluoride (PVDF) membranes (Bio-Rad). The membranes were blocked for 2 h in 5% nonfat milk and then incubated with diluted primary antibodies for 2 h at room temperature or overnight at 4°C. Primary antibodies against the FLAG tag (catalog no. SI-A8592; Sigma), Sp1 (catalog no. CS200631; Millipore), Sp3 (catalog no. sc-365220; Santa Cruz), NF1 (catalog no. sc-5567; Santa Cruz), C/EBPα (catalog no. sc-9314; Santa Cruz), GFP (catalog no. G1544; Sigma), RBP-Jκ (catalog no. ab25949; Abcam), and actin (catalog no. sc-47778; Santa Cruz) were obtained commercially. The rabbit polyclonal antibody against ORF50 was generated in our laboratory using bacterially produced ORF50 protein (aa 1 to 590) as an antigen. Anti-rabbit or anti-mouse immunoglobulin G antibody conjugated to horseradish peroxidase was used as the secondary antibody. The enhanced chemiluminescence system was used for the detection of antibody-antigen complexes.

### Coimmunoprecipitation.

Plasmids, including pEGFP-C2, pCMV-GFP-ORF50(1–590), or pCMV-GFP-ORF50(1–490), were transfected into 293T cells for 24 h. The transfected cells were collected and lysed in immunoprecipitation assay buffer containing 50 mM Tris-Cl (pH 7.6), 100 mM NaCl, 1 mM EDTA, 0.5% Triton X-100, 0.5% NP-40, 0.1% sodium deoxycholate, and 1 mM phenylmethylsulfonyl fluoride. The prepared protein lysates were immunoprecipitated by incubation with GFP-Trap_MA beads (catalog no. gtma-100; Chromotek), and the immunoprecipitates were then probed with specific antibodies.

### Statistical analysis.

Reporter assays were repeated independently at least three times. To demonstrate reproducibility, some EMSAs were repeated three times. All summary data are expressed as means ± standard errors. Statistical analysis was performed using SPSS (Statistical Package for the Social Sciences) software version 18.0. Student’s *t* test was used to evaluate the significance of differences between groups. A *P* value of <0.05 was considered statistically significant.
